# Improved Approach for the Maximum Entropy Deconvolution Problem

**DOI:** 10.3390/e23050547

**Published:** 2021-04-28

**Authors:** Shay Shlisel, Monika Pinchas

**Affiliations:** Department of Electrical and Electronic Engineering, Ariel University, Ariel 40700, Israel; shayshlisel@gmail.com

**Keywords:** maximum entropy, deconvolution, blind equalization, edgeworth expansion, Generalized Gaussian Distribution (GGD), Laplace integral

## Abstract

The probability density function (pdf) valid for the Gaussian case is often applied for describing the convolutional noise pdf in the blind adaptive deconvolution problem, although it is known that it can be applied only at the latter stages of the deconvolution process, where the convolutional noise pdf tends to be approximately Gaussian. Recently, the deconvolutional noise pdf was approximated with the Edgeworth Expansion and with the Maximum Entropy density function for the 16 Quadrature Amplitude Modulation (QAM) input but no equalization performance improvement was seen for the hard channel case with the equalization algorithm based on the Maximum Entropy density function approach for the convolutional noise pdf compared with the original Maximum Entropy algorithm, while for the Edgeworth Expansion approximation technique, additional predefined parameters were needed in the algorithm. In this paper, the Generalized Gaussian density (GGD) function and the Edgeworth Expansion are applied for approximating the convolutional noise pdf for the 16 QAM input case, with no need for additional predefined parameters in the obtained equalization method. Simulation results indicate that improved equalization performance is obtained from the convergence time point of view of approximately 15,000 symbols for the hard channel case with our new proposed equalization method based on the new model for the convolutional noise pdf compared to the original Maximum Entropy algorithm. By convergence time, we mean the number of symbols required to reach a residual inter-symbol-interference (ISI) for which reliable decisions can be made on the equalized output sequence.

## 1. Introduction

In this paper, the blind adaptive deconvolution problem (blind adaptive equalizer) is considered, where we observe the output of an unknown linear system (channel) from which we want to recover its input, using an adaptive blind equalizer (adaptive linear filter) [1,2,3,4,5,6]. The linear system (channel) is often modeled as a finite impulse response (FIR) filter. Since the channel coefficients are unknown, the equalizer’s coefficients used in the deconvolution process are only approximated values leading to an error signal that is added to the source signal at the output of the deconvolution process. In the following, we define this error signal throughout the paper as the convolutional noise. The Gaussian pdf is often applied in the literature [1,7,8,9,10,11], for approximating the convolutional noise pdf in calculating the conditional expectation of the source input given the equalized output sequence, based on Bayes rules. However, according to [8], the convolutional noise pdf tends approximately to a Gaussian pdf only at the latter stages of the iterative deconvolution process, where the equalizer has converged to a relative low residual ISI (where the convolutional noise is relative low). In the early stages of the iterative deconvolution process, the ISI is typically large with the result that the input sequence and the convolutional noise sequence are strongly correlated and the convolutional noise pdf is more uniform than Gaussian [8,12]. Recently [3,4], the convolutional noise pdf was approximated with the Maximum Entropy density approximation technique [1,2,13,14] with Lagrange multipliers up to order four and with the Edgeworth Expansion series [15,16] up to order six, to obtain the conditional expectation of the source signal (16 QAM input case), given the equalized output via Bayes rules. However, as demonstrated in [3], the blind adaptive equalization algorithm with the closed-form approximated expression for the conditional expectation based on approximating the convolutional noise pdf with the Maximum Entropy density approximation technique, achieved for the hard channel case (named the channel4 case in [3]), the same equalization performance from the residual ISI and convergence time point of view compared with the original blind adaptive equalization algorithm [1] where the convolutional noise pdf was approximated with the Gaussian pdf to obtain the closed-form approximated expression for the conditional expectation of the source signal given the equalized output via Bayes rules. The equalization performance obtained with the Edgeworth Expansion approach [4] was indeed improved compared with the original blind adaptive equalization algorithm [1] where the convolutional noise pdf was approximated with the Gaussian pdf to obtain the closed-form approximated expression for the conditional expectation of the source signal given the equalized output via Bayes rules. However, this equalization method [4] needed two additional predefined parameters (additional to the predefined step-size parameter involved in the equalizer’s coefficients update mechanism) in the algorithm. These two additional predefined parameters where used in the approximation for the fourth and sixth moment of the convolutional noise. Since the convolutional noise is channel dependent, the various moments of the convolutional noise are also channel dependent which lead also to the two additional predefined parameters in [4] to be channel dependent. As it was already implied earlier, the shape of the convolutional noise pdf changes during the iterative deconvolution process. Thus, if we could have an approximation for the convolutional pdf that is close to optimality, we could have a closed-form approximated expression for the conditional expectation of the source signal given the equalized output via Bayes rules that may lead to improved equalization performance from the residual ISI and convergence time point of view compared to existing methods based on the closed-form approximated conditional expectation expression [1,3,4]. According to [17,18,19], the GGD provides a flexible and suitable tool for data modeling and simulation. The GGD [17,18] is based on a shape parameter that changes the pdf which may have a Laplacian, or double exponential distribution, a Gaussian distribution or a uniform distribution for a shape parameter equal to one, two and infinity respectively. The shape of the convolutional noise pdf changes as a function of the residual ISI. Thus, in order to apply the GGD for the convolutional noise pdf approximation task, the shape parameter related to the GGD presentation must be a function of the residual ISI. Recently [20], the shape parameter related to the GGD presentation [17,18] was given as a function of the residual ISI.

In this paper, we deal with the 16QAM input case where we use the GGD presentation [17,18] with the results obtained from [20], to approximate the convolutional noise pdf involved in the calculation of the closed-form approximated expression for the conditional expectation of the source signal given the equalized output via Bayes rules. Since the shape parameter related to the GGD presentation [17,18] may have also fractional values during the iterative deconvolution process, the integral involved in the conditional expectation calculation may not lead to a closed-form approximated expression. Thus, we use in this work the Edgeworth Expansion series [15,16] up to order six for approximating the GGD presentation applicable for the convolutional noise pdf where the fourth and sixth moments of the convolutional noise sequence are approximated with the GGD technique [17,18]. By applying the GGD [17,18], the Edgeworth Expansion [15,16] and the results from [20] (the relationship between the shape parameter and the residual ISI), a new closed-form approximated expression is proposed for the conditional expectation of the source signal given the equalized output via Bayes rules that has no need for additional predefined parameters in the obtained equalization method as is the case in [4]. Simulation results indicate that with our new proposed equalization method based on the new model for the convolutional noise pdf we have:

- Improved equalization performance from the convergence time point of view for the easy [6] as well as for the hard channel case, compared to the original Maximum Entropy algorithm [1]. The improvement in the convergence time for the hard channel case is approximately of 15,000 symbols while for the easy channel case the improvement in the convergence time is approximately of 250 symbols. In both cases we may say that the improvement in the convergence time is approximately third of the convergence time of the original Maximum Entropy algorithm [1].
- Based on [3], the blind adaptive equalization algorithm with the closed-form approximated expression for the conditional expectation based on approximating the convolutional noise pdf with the Maximum Entropy density approximation technique, achieved for the hard channel case, the same equalization performance from the residual ISI and convergence time point of view as was achieved with the original Maximum Entropy algorithm [1]. Thus, the improvement in the convergence time with our new proposed method compared with the algorithm in [3] is also approximately of 15,000 symbols for the hard channel case.
- The new proposed equalization method does not need additional predefined parameters (additional to the predefined step-size parameter involved in the equalizer’s coefficients update mechanism) in the algorithm in order to get improved convergence time compared to the original Maximum Entropy algorithm [1], as does the algorithm in [4] where the convolutional noise pdf was approximated with the Edgeworth Expansion series.
- For the easy channel case and SNR of 26 dB, the new proposed equalization method has improved equalization performance from the residual ISI and convergence time point of view compared to the recently proposed methods [2,5] which are versions of the original Maximum Entropy algorithm [1]. From the residual ISI point of view, the improvement is approximately 4 dB while the improvement in the convergence time is approximately third of the convergence time achieved by the equalization methods presented in [2,5].

The paper is organized as follows—after having described the system under consideration in Section 2, the systematic way for obtaining the closed-form approximated expression for the conditional expectation of the source signal given the equalized output via Bayes rules based on the GGD and Edgeworth Expansion series is given in Section 3. In Section 4 we introduce our simulation results. Finally, the conclusion is presented in Section 5.

## 2. System Description

**Figure 1:**
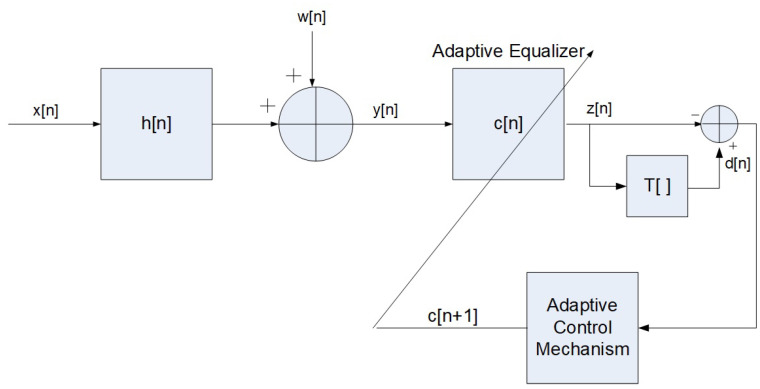
A block diagram for baseband communication transmission.

In the following (Figure 1), we recall the system under consideration used in [1,3,4], where we apply the same assumptions made in [1,3,4]:

- The input sequence x[n] is a 16QAM source (a modulation using ± {1,3} levels for in-phase and quadrature components) which can be written as $x\left[n\right]=x_{1}\left[n\right]+jx_{2}\left[n\right]$ where $x_{1}\left[n\right]$ and $x_{2}\left[n\right]$ are the real and imaginary parts of x[n], respectively. $x_{1}\left[n\right]$ and $x_{2}\left[n\right]$ are independent and E[x[n]]=0 (where E[·] denotes the expectation operator on (·)).
- The unknown channel h[n] is a possibly non-minimum phase linear time-invariant filter in which the transfer function has no “deep zeros”; namely, the zeros lie sufficiently far from the unit circle.
- The filter c[n] is a tap-delay line.
- The channel noise w[n] is an additive Gaussian white noise.
- The function T[·] is a memoryless nonlinear function that satisfies the additivity condition:
  (1) $$T\left[z_{1}\left[n\right]+jz_{2}\left[n\right]\right]=T\left[z_{1}\left[n\right]\right]+jT\left[z_{2}\left[n\right]\right],$$
  where $z_{1}\left[n\right]$, $z_{2}\left[n\right]$ are the real and imaginary parts of the equalized output, respectively.

The input to the equalizer is given by:

(2) y[n]=x[n]∗h[n]+w[n],

where “∗” stands for the convolutional operation. Based on (2), the equalized output is obtained via:

(3) $$z\left[n\right]=y\left[n\right]*c\left[n\right]=x\left[n\right]*\tilde{s}\left[n\right]+\tilde{w}\left[n\right]=x\left[n\right]+p\left[n\right]+\tilde{w}\left[n\right],$$

where

(4) $$\begin{array}{c} \tilde{s}\left[n\right]=c\left[n\right]*h\left[n\right]=\delta \left[n\right]+\xi \left[n\right]\\ p\left[n\right]=x\left[n\right]*\xi \left[n\right], \end{array}$$

where ξ[n] stands for the difference (error) between the ideal and the used value for c[n] following (6), δ is the Kronecker delta function, $\tilde{w}\left[n\right]=w\left[n\right]*c\left[n\right]$ and p[n] is the convolutional noise. The ISI is expressed by:

(5) $$ISI=\frac{\sum_{\tilde{m}}|\tilde{s}\left[\tilde{m}\right]{|}^{2}-{\left|\tilde{s}\right|}_{max}^{2}}{|\tilde{s}{|}_{max}^{2}},$$

where $|\tilde{s}{|}_{max}$ is the component of $\tilde{s}$, given in (4), having the maximal absolute value. The function T[z[n]] is an estimation to x[n] where d[n]=T[z[n]]. The equalizer is updated according to:

(6) $$\underline{c}\left[n+1\right]=\underline{c}\left[n\right]+\mu \left(T\left[z\left[n\right]\right]-z\left[n\right]\right),{\underline{y}}^{*}\left[n\right]$$

where ${\left(\cdot \right)}^{*}$ is the conjugate operation on (·), μ is the step size parameter and $\underline{c}\left[n\right]$ is the equalizer vector, where the input vector is $\underline{y}\left[n\right]={\left[y\left[n\right]...y\left[n-N+1\right]\right]}^{T}$. The operator ${()}^{T}$ denotes the transpose of the function (), and *N* is the equalizer’s tap length.

## 3. GGD Based Closed-Form Approximated Expression for the Conditional Expectation

In this section, we present a systematic approach for obtaining the conditional expectation (E[x[n]|z[n]]) based on approximating the convolutional noise pdf with the GGD [17,18] and Edgeworth Expansion [15,16] presentations. For simplicity, we use in the following, *x*, *y*, *p* for x[n], y[n] and p[n], respectively.

**Theorem** **1.** *For the noiseless and 16QAM input case, the closed-form approximated expression for the conditional expectation (E[x|z]) is given by:*

(7) $$\begin{array}{c} E\left[x|z\right]≃\frac{u_{1}}{f_{1}}+j\frac{u_{2}}{f_{2}},\\ wherefor\;i=1,2\;\;K=4\;\;and\;k=2,4\\ u_{i}=z_{i}+\frac{1}{2}\left(2\left(3T-15V+1\right)\sum_{k=2}^{K}kz_{i}^{k-1}\lambda_{k}-\left(\frac{12T}{\sigma_{p_{i}}^{2}}-\frac{90V}{\sigma_{p_{i}}^{2}}\right)z_{i}+\right.\\ \left.\left(3T-15V+1\right)z_{i}\left({\left(\sum_{k=2}^{K}kz_{i}^{k-1}\lambda_{k}\right)}^{2}+\sum_{k=2}^{K}kz_{i}^{k-2}\lambda_{k}\left(k-1\right)\right)\right)\left(\sigma_{z_{i}}^{2}-\sigma_{x_{i}}^{2}\right)+\\ \frac{1}{8}\left(4\left(3T-15V+1\right){\left(\sum_{k=2}^{K}\frac{k}{z_{i}}z_{i}^{k}\lambda_{k}\right)}^{3}+\right.\\ \left.4\left(3T-15V+1\right)\sum_{k=2}^{K}\frac{1}{z_{i}^{3}}\left(k^{3}z_{i}^{k}\lambda_{k}-3k^{2}z_{i}^{k}\lambda_{k}+2kz_{i}^{k}\lambda_{k}\right)-\right.\\ \left.12\left(\frac{12T}{\sigma_{p_{i}}^{2}}-\frac{90V}{\sigma_{p_{i}}^{2}}\right)\sum_{k=2}^{K}\frac{k}{z_{i}}z_{i}^{k}\lambda_{k}+\left(\frac{24T}{\sigma_{p_{i}}^{4}}-\frac{360V}{\sigma_{p_{i}}^{4}}\right)z+\right.\\ \left.3\left(3T-15V+1\right)z_{i}{\left(\sum_{k=2}^{K}\frac{1}{z_{i}^{2}}\left(k^{2}z_{i}^{k}\lambda_{k}-kz_{i}^{k}\lambda_{k}\right)\right)}^{2}-\right.\\ \left.6\left(\frac{12T}{\sigma_{p_{i}}^{2}}-\frac{90V}{\sigma_{p_{i}}^{2}}\right)z_{i}{\left(\sum_{k=2}^{K}\frac{k}{z_{i}}z_{i}^{k}\lambda_{k}\right)}^{2}+\left(3T-15V+1\right)z_{i}{\left(\sum_{k=2}^{K}\frac{k}{z_{i}}z_{i}^{k}\lambda_{k}\right)}^{4}+\right.\\ \left.12\left(3T-15V+1\right)\left(\sum_{k=2}^{K}\frac{1}{z_{i}^{2}}\left(k^{2}z_{i}^{k}\lambda_{k}-kz_{i}^{k}\lambda_{k}\right)\right)\sum_{k=2}^{K}\frac{k}{z_{i}}z_{i}^{k}\lambda_{k}+\right.\\ \left.\left(3T-15V+1\right)z_{i}\sum_{k=2}^{K}\frac{1}{z_{i}^{4}}\left(11k^{2}z_{i}^{k}\lambda_{k}-6k^{3}z_{i}^{k}\lambda_{k}+k^{4}z_{i}^{k}\lambda_{k}-6kz_{i}^{k}\lambda_{k}\right)-\right.\\ \left.6\left(\frac{12T}{\sigma_{p_{i}}^{2}}-\frac{90V}{\sigma_{p_{i}}^{2}}\right)z_{i}\sum_{k=2}^{K}\frac{1}{z_{i}^{2}}\left(k^{2}z_{i}^{k}\lambda_{k}-kz_{i}^{k}\lambda_{k}\right)+\right.\\ \left.4\left(3T-15V+1\right)z_{i}\left(\sum_{k=2}^{K}\frac{1}{z_{i}^{3}}\left(k^{3}z_{i}^{k}\lambda_{k}-3k^{2}z_{i}^{k}\lambda_{k}+2kz_{i}^{k}\lambda_{k}\right)\right)\sum_{k=2}^{K}\frac{k}{z_{i}}z_{i}^{k}\lambda_{k}+\right.\\ \left.6\left(3T-15V+1\right)z_{i}\left(\sum_{k=2}^{K}\frac{1}{z_{i}^{2}}\left(k^{2}z_{i}^{k}\lambda_{k}-kz_{i}^{k}\lambda_{k}\right)\right){\left(\sum_{k=2}^{K}\frac{k}{z_{i}}z_{i}^{k}\lambda_{k}\right)}^{2}\right){\left(\sigma_{z_{i}}^{2}-\sigma_{x_{i}}^{2}\right)}^{2}\\ and\\ f_{i}=1+\frac{1}{2}\left({\left(\sum_{k=2}^{K}kz_{i}^{k-1}\lambda_{k}\right)}^{2}+\sum_{k=2}^{K}kz_{i}^{k-2}\lambda_{k}\left(k-1\right)\right)\left(\sigma_{z_{i}}^{2}-\sigma_{x_{i}}^{2}\right)+\\ \frac{1}{8}\left({\left(\sum_{k=2}^{K}kz_{i}^{k-1}\lambda_{k}\right)}^{4}+6{\left(\sum_{k=2}^{K}kz_{i}^{k-1}\lambda_{k}\right)}^{2}\sum_{k=2}^{K}kz_{i}^{k-2}\lambda_{k}\left(k-1\right)+\right.\\ \left.4\left(\sum_{k=2}^{K}kz_{i}^{k-3}\lambda_{k}\left(k-1\right)\left(k-2\right)\right)\sum_{k=2}^{K}kz_{i}^{k-1}\lambda_{k}+\right.\\ \left.3{\left(\sum_{k=2}^{K}kz_{i}^{k-2}\lambda_{k}\left(k-1\right)\right)}^{2}+\sum_{k=2}^{K}kz_{i}^{k-4}\lambda_{k}\left(k-1\right)\left(k-2\right)\left(k-3\right)\right){\left(\sigma_{z_{i}}^{2}-\sigma_{x_{i}}^{2}\right)}^{2},\\ with\\ \sigma_{p_{i}}^{2}=\sigma_{z_{i}}^{2}-\sigma_{x_{i}}^{2}, \end{array}$$

*where*

(8) $$\begin{array}{c} T=\left(\frac{\frac{\Gamma (1/\beta )}{\Gamma^{2}\left(3/\beta \right)}\Gamma \left(5/\beta \right)-3}{4!}\right);\;\;\;V=\left(\frac{\frac{\Gamma^{2}\left(1/\beta \right)}{\Gamma^{3}\left(3/\beta \right)}\Gamma \left(7/\beta \right)-15\frac{\Gamma (1/\beta )}{\Gamma^{2}\left(3/\beta \right)}\Gamma \left(5/\beta \right)+30}{6!}\right), \end{array}$$

*and where* Γ *is the Gamma function and* β *is given by [20]:*

(9) $$\begin{array}{c} \beta ≅-1.1938\times 10^{-5}{\left(ISI_{dB}\right)}^{4}-7.3370\times 10^{-4}{\left(ISI_{dB}\right)}^{3}-\\ 0.0146{\left(ISI_{dB}\right)}^{2}-0.0693\left(ISI_{dB}\right)+2.6266\\ ISI_{dB}=10log_{10}\left(ISI\right). \end{array}$$

*In this work the* ISI *is expressed as:*

(10) $$ISI=\frac{\sigma_{p_{1}}^{2}}{\sigma_{x_{1}}^{2}}$$

*and the Lagrange multipliers for k=2,4 ($\lambda_{2}$, $\lambda_{4}$) are calculated according to [1]:*

(11) $$\begin{array}{c} 1+4\lambda_{2}m_{2}+8\lambda_{4}m_{4}=0\\ 3m_{2}+8\lambda_{4}m_{6}+4\lambda_{2}m_{4}=0, \end{array}$$

*where*

(12) $$m_{k}=E\left[x_{1}^{k}\right].$$

**Proof of Theorem 1.**  For the two independent quadrature carrier case where the 16QAM modulation is a special case of it, the conditional expectation (E[x|z]) can be given according to [9] as:

(13) $$E\left[x|z\right]=E\left[x_{1}|z_{1}\right]+jE\left[x_{2}|z_{2}\right].$$

Thus, real and imaginary parts of the data are to be estimated separately on the basis of the real and imaginary parts of the equalizer’s output sequence. For the noiseless case, (3) may be written as:

(14) p=z−x.

In the following, we denote $p_{1}$ and $p_{2}$ as the real and imaginary parts of *p*. Based on (14) and under the assumption that the blind adaptive equalizer leaves the system with a relative low residual ISI for which the input signal *x* and the convolutional noise signal *p* can be considered as independent [8], we may write for the 16QAM modulation case:

(15) $$\begin{array}{c} \sigma_{p}^{2}=\sigma_{z}^{2}-\sigma_{x}^{2}=2\sigma_{p_{1}}^{2}=2\sigma_{p_{2}}^{2}=2\sigma_{z_{1}}^{2}-2\sigma_{x_{1}}^{2}=2\sigma_{z_{2}}^{2}-2\sigma_{x_{2}}^{2}\\ ⇓\\ \sigma_{p_{1}}^{2}=\sigma_{z_{1}}^{2}-\sigma_{x_{1}}^{2}. \end{array}$$

Based on (3), the variance of the real part of the equalized output signal $\sigma_{z_{1}}^{2}$ can be written for the noiseless case as:

(16) $$\sigma_{z_{1}}^{2}=\sigma_{x_{1}}^{2}\sum_{\tilde{m}}{\left|{\tilde{s}}_{\tilde{m}}\left[n\right]\right|}^{2}.$$

Next, based on (16), (15) and (5) we may write:

(17) $$\begin{array}{c} 2\sigma_{p_{1}}^{2}=2\sigma_{x_{1}}^{2}\sum_{\tilde{m}}{\left|{\tilde{s}}_{\tilde{m}}\left[n\right]\right|}^{2}-2\sigma_{x_{1}}^{2}=2\sigma_{x_{1}}^{2}\left(\sum_{\tilde{m}}{\left|{\tilde{s}}_{\tilde{m}}\left[n\right]\right|}^{2}-1\right)\\ ⇓\\ 2\sigma_{p_{1}}^{2}=2\sigma_{x_{1}}^{2}ISI\;\;for|\tilde{s}{|}_{max}=1\\ ⇓\\ \frac{\sigma_{p_{1}}^{2}}{\sigma_{x_{1}}^{2}}=ISI\;\;for|\tilde{s}{|}_{max}=1. \end{array}$$

Next, we show the systematic approach for calculating the conditional expectation $E[x_{1}|z_{1}]$. The conditional expectation $E[x_{1}|z_{1}]$ is defined by:

(18) $$\begin{array}{c} E\left[x_{1}|z_{1}\right]=\int_{-\infty }^{+\infty }x_{1}f_{x_{1}|z_{1}}\left(x_{1}|z_{1}\right)dx_{1}, \end{array}$$

where $f_{x_{1}|z_{1}}\left(x_{1}|z_{1}\right)$ is the conditional pdf. Based on Bayes rules we may write:

(19) $$\begin{array}{c} f_{x_{1}|z_{1}}\left(x_{1}|z_{1}\right)=\frac{f_{z_{1}|x_{1}}\left(z_{1}|x_{1}\right)f_{x_{1}}\left(x_{1}\right)}{f_{z_{1}}\left(z_{1}\right)}=\\ \frac{f_{z_{1}|x_{1}}\left(z_{1}|x_{1}\right)f_{x_{1}}\left(x_{1}\right)}{\int_{-\infty }^{+\infty }f_{z_{1}|x_{1}}\left(z_{1}|x_{1}\right)f_{x_{1}}\left(x_{1}\right)dx_{1}}. \end{array}$$

Now, by substituting (19) into (18) we obtain:

(20) $$\begin{array}{c} E\left[x_{1}|z_{1}\right]=\frac{\int_{-\infty }^{+\infty }x_{1}f_{z_{1}|x_{1}}\left(z_{1}|x_{1}\right)f_{x_{1}}\left(x_{1}\right)dx_{1}}{\int_{-\infty }^{+\infty }f_{z_{1}|x_{1}}\left(z_{1}|x_{1}\right)f_{x_{1}}\left(x_{1}\right)dx_{1}}. \end{array}$$

As was already mentioned earlier in this paper, we would like to use the GGD [17,18] presentation for approximating the real part of the convolutional noise pdf. Thus, based on the GGD [17,18] the real part of the convolutional noise pdf is approximately given by:

(21) $$f_{p_{1}}\left(p_{1}\right)≃\frac{1}{2\Gamma \left(1+\frac{1}{\beta }\right)B\left(\beta ,\sigma \right)}\exp\left(-|\frac{p_{1}}{B\left(\beta ,\sigma \right)}{|}^{\beta }\right),$$

with

(22) $$B\left(\beta ,\sigma \right)={\left(\frac{\sigma_{p_{1}}^{2}\Gamma \left(\frac{1}{\beta }\right)}{\Gamma \left(\frac{3}{\beta }\right)}\right)}^{\frac{1}{2}},$$

where β is defined as the shape parameter of the pdf presentation. Thus, based on [17,18], (21) and (14), the conditional pdf $f_{z_{1}|x_{1}}\left(z_{1}|x_{1}\right)$ can be expressed by:

(23) $$f_{z_{1}|x_{1}}\left(z_{1}|x_{1}\right)≃\frac{1}{2\Gamma \left(1+\frac{1}{\beta }\right)B\left(\beta ,\sigma \right)}\exp\left(-|\frac{z_{1}-x_{1}}{B\left(\beta ,\sigma \right)}{|}^{\beta }\right).$$

Following [1,3,4], we use the Maximum Entropy density approximation technique [13,14] with Lagrange multipliers up to order four, for approximating the pdf of the real part input sequence:

(24) $$f_{x_{1}}\left(x_{1}\right)≃A\exp\left(\lambda_{2}x_{1}^{2}+\lambda_{4}x_{1}^{4}\right),$$

where $\lambda_{2}$ and $\lambda_{4}$ are the Lagrange multipliers and *A* is a constant. Next, by substituting (23) and (24) into (20), some problems are noticed in carrying out the integrals involved in (20) for achieving a closed-form approximated expression for the conditional expectation $E[x_{1}|z_{1}]$ due to the fact that the shape parameter β is a changing parameter during the iterative blind deconvolution process that may have also non integer values. Thus, to overcome the problem, we apply the Edgeworth Expansion series [15,16] up to order six for approximating the real part of the convolutional noise pdf where the higher moments of the convolutional noise sequence are calculated via the GGD [17,18] technique:

(25) $$\begin{array}{c} f_{p_{1}}\left(p_{1}\right)≃\frac{\exp\left(-\frac{p_{1}^{2}}{2\sigma_{p_{1}}^{2}}\right)}{\sqrt{2\pi }\sigma_{p_{1}}}\left[1+\left(\frac{E\left[p_{1}^{4}\right]-3{\left(\sigma_{p_{1}}^{2}\right)}^{2}}{4!{\left(\sigma_{p_{1}}^{2}\right)}^{2}}\right)\left(\frac{p_{1}^{4}}{{\left(\sigma_{p_{1}}^{2}\right)}^{2}}-\frac{6p_{1}^{2}}{\sigma_{p_{1}}^{2}}+3\right)+\right.\\ \left.\left(\frac{E\left[p_{1}^{6}\right]-15\sigma_{p_{1}}^{2}E\left[p_{1}^{4}\right]+30{\left(\sigma_{p_{1}}^{2}\right)}^{3}}{6!{\left(\sigma_{p_{1}}^{2}\right)}^{3}}\right)\left(\frac{p_{1}^{6}}{{\left(\sigma_{p_{1}}^{2}\right)}^{3}}-\frac{15p_{1}^{4}}{{\left(\sigma_{p_{1}}^{2}\right)}^{2}}+\frac{45p_{1}^{2}}{\sigma_{p_{1}}^{2}}-15\right)\right]\\ \mathrm{with}\\ E\left[p_{1}^{6}\right]={\left[\frac{\sigma_{p_{1}}^{2}\Gamma \left(\frac{1}{\beta }\right)}{\Gamma \left(\frac{3}{\beta }\right)}\right]}^{3}\frac{\Gamma \left(\frac{7}{\beta }\right)}{\Gamma \left(\frac{1}{\beta }\right)};\;\;\;E\left[p_{1}^{4}\right]={\left[\frac{\sigma_{p_{1}}^{2}\Gamma \left(\frac{1}{\beta }\right)}{\Gamma \left(\frac{3}{\beta }\right)}\right]}^{2}\frac{\Gamma \left(\frac{5}{\beta }\right)}{\Gamma \left(\frac{1}{\beta }\right).} \end{array}$$

Thus, based on the Edgeworth Expansion series technique [15,16] and (25) we have:

(26) $$\begin{array}{c} f_{z_{1}|x_{1}}\left(z_{1}|x_{1}\right)≃\frac{\exp\left(-\frac{{\left(z_{1}-x_{1}\right)}^{2}}{2\sigma_{p_{1}}^{2}}\right)}{\sqrt{2\pi }\sigma_{p_{1}}}\left[1+\left(\frac{E\left[p_{1}^{4}\right]-3{\left(\sigma_{p_{1}}^{2}\right)}^{2}}{4!{\left(\sigma_{p_{1}}^{2}\right)}^{2}}\right)\left(\frac{{\left(z_{1}-x_{1}\right)}^{4}}{{\left(\sigma_{p_{1}}^{2}\right)}^{2}}-\frac{6{\left(z_{1}-x_{1}\right)}^{2}}{\sigma_{p_{1}}^{2}}+3\right)+\right.\\ \left.\left(\frac{E\left[p_{1}^{6}\right]-15\sigma_{p_{1}}^{2}E\left[p_{1}^{4}\right]+30{\left(\sigma_{p_{1}}^{2}\right)}^{3}}{6!{\left(\sigma_{p_{1}}^{2}\right)}^{3}}\right)\left(\frac{{\left(z_{1}-x_{1}\right)}^{6}}{{\left(\sigma_{p_{1}}^{2}\right)}^{3}}-\frac{15{\left(z_{1}-x_{1}\right)}^{4}}{{\left(\sigma_{p_{1}}^{2}\right)}^{2}}+\frac{45{\left(z_{1}-x_{1}\right)}^{2}}{\sigma_{p_{1}}^{2}}-15\right)\right] \end{array}$$

with $E\left[p_{1}^{6}\right]$ and $E\left[p_{1}^{4}\right]$ given in (25). Now, substituting (26) and (24) into (20) yields:

(27) $$E\left[x_{1}|z_{1}\right]≃\frac{\int_{-\infty }^{\infty }g_{1}\left(x_{1}\right)\exp\left(-\Psi \left(x_{1}\right)/\rho \right)dx_{1}}{\int_{-\infty }^{\infty }g\left(x_{1}\right)\exp\left(-\Psi \left(x_{1}\right)/\rho \right)dx_{1}},$$

where

(28) $$\begin{array}{c} \rho =2\sigma_{p_{1}}^{2};\;\;\Psi \left(x_{1}\right)={\left(z_{1}-x_{1}\right)}^{2};\;\;g_{1}\left(x_{1}\right)=x_{1}g\left(x_{1}\right);\\ g\left(x_{1}\right)=\tilde{g}\left(x_{1}\right)\left[1+\left(\frac{E\left[p_{1}^{4}\right]-3{\left(\sigma_{p_{1}}^{2}\right)}^{2}}{4!{\left(\sigma_{p_{1}}^{2}\right)}^{2}}\right)\left(\frac{{\left(z_{1}-x_{1}\right)}^{4}}{{\left(\sigma_{p_{1}}^{2}\right)}^{2}}-\frac{6{\left(z_{1}-x_{1}\right)}^{2}}{\sigma_{p_{1}}^{2}}+3\right)+\right.\\ \left.\left(\frac{E\left[p_{1}^{6}\right]-15\sigma_{p_{1}}^{2}E\left[p_{1}^{4}\right]+30{\left(\sigma_{p_{1}}^{2}\right)}^{3}}{6!{\left(\sigma_{p_{1}}^{2}\right)}^{3}}\right)\left(\frac{{\left(z_{1}-x_{1}\right)}^{6}}{{\left(\sigma_{p_{1}}^{2}\right)}^{3}}-\frac{15{\left(z_{1}-x_{1}\right)}^{4}}{{\left(\sigma_{p_{1}}^{2}\right)}^{2}}+\frac{45{\left(z_{1}-x_{1}\right)}^{2}}{\sigma_{p_{1}}^{2}}-15\right)\right]\\ \tilde{g}\left(x_{1}\right)=\exp\left(\lambda_{2}x_{1}^{2}+\lambda_{4}x_{1}^{4}\right). \end{array}$$

In order to obtain closed-form expressions for the integrals involved in (27), the Laplace’s method [21] is applied as was also done in [1,3,4]. According to [21], the Laplace’s method is a general technique for obtaining the asymptotic behavior as ρ→0 of integrals in which the large parameter 1/ρ appears in the exponent. The main idea of Laplace’s method is: if the continues function $\Psi (x_{1})$ has its minimum at $x_{0}$ which is between infinity and minus infinity, then it is only the immediate neighborhood of $x_{1}=x_{0}$ that contributes to the full asymptotic expansion of the integral for large 1/ρ. Thus, according to [1,3,4,21]:

(29) $$\begin{array}{c} \int_{-\infty }^{\infty }g\left(x_{1}\right)\exp\left(-\Psi \left(x_{1}\right)/\rho \right)dx_{1}≃\\ \exp\left(-\frac{\Psi (x_{0})}{\rho }\right)\sqrt{\frac{2\pi \rho }{\Psi^{{}^{\prime \prime }}\left(x_{0}\right)}}\left(g\left(x_{0}\right)+\frac{g^{{}^{\prime \prime }}\left(x_{0}\right)}{2}\frac{\rho }{\Psi^{{}^{\prime \prime }}\left(x_{0}\right)}+\frac{g^{{}^{\prime \prime \prime \prime }}\left(x_{0}\right)}{8}{\left(\frac{\rho }{\Psi^{{}^{\prime \prime }}\left(x_{0}\right)}\right)}^{2}+O{\left(\frac{\rho }{\Psi^{{}^{\prime \prime }}\left(x_{0}\right)}\right)}^{3}\right), \end{array}$$

(30) $$\begin{array}{c} \int_{-\infty }^{\infty }g_{1}\left(x_{1}\right)\exp\left(-\Psi \left(x_{1}\right)/\rho \right)dx_{1}≃\\ \exp\left(-\frac{\Psi (x_{0})}{\rho }\right)\sqrt{\frac{2\pi \rho }{\Psi^{{}^{\prime \prime }}\left(x_{0}\right)}}\left(g_{1}\left(x_{0}\right)+\frac{g_{1}^{{}^{\prime \prime }}\left(x_{0}\right)}{2}\frac{\rho }{\Psi^{{}^{\prime \prime }}\left(x_{0}\right)}+\frac{g_{1}^{{}^{\prime \prime \prime \prime }}\left(x_{0}\right)}{8}{\left(\frac{\rho }{\Psi^{{}^{\prime \prime }}\left(x_{0}\right)}\right)}^{2}+O{\left(\frac{\rho }{\Psi^{{}^{\prime \prime }}\left(x_{0}\right)}\right)}^{3}\right), \end{array}$$

where ${\left(\right)}^{\prime \prime }$ and ${\left(\right)}^{\prime \prime \prime \prime }$ stand for the second and fourth derivative of (), respectively, $O\left(x\right)$ is defined as $lim_{x\to 0}O\left(x\right)/x=r_{const}$ and $r_{const}$ is a constant. The expressions for $\Psi^{{}^{\prime \prime }}\left(x_{0}\right)$ and $x_{0}$ are given by:

(31) $$\begin{array}{c} \Psi^{{}^{\prime }}\left(x_{1}\right)=-2\left(z_{1}-x_{1}\right);\;\;\Psi^{{}^{\prime \prime }}\left(x_{1}\right)=2\Rightarrow \Psi^{{}^{\prime }}\left(x_{0}\right)=2\\ \Psi^{{}^{\prime }}\left(x_{0}\right)=-2\left(z_{1}-x_{0}\right)=0\Rightarrow x_{0}=z_{1}. \end{array}$$

Now, by substituting (29) and (30) into (27), dividing both the numerator and denominator by the function $\tilde{g}\left(z_{1}\right)$ given in (28) with $z_{1}$ instead of $x_{1}$, $x_{0}=z_{1}$, $\Psi^{{}^{\prime }}\left(x_{0}\right)=2$ from (31), $\rho =2\sigma_{p_{1}}^{2}$ from (28) and $\sigma_{p_{1}}^{2}=\sigma_{z_{1}}^{2}-\sigma_{x_{1}}^{2}$ from (15) we obtain:

(32) $$\begin{array}{c} E\left[x_{1}|z_{1}\right]≃\frac{{E\left[x_{1}|z_{1}\right]}_{up}}{{E\left[x_{1}|z_{1}\right]}_{down}} \end{array}$$

(33) $$\begin{array}{c} {E\left[x_{1}|z_{1}\right]}_{up}=z_{1}+\left(g_{1}^{{}^{\prime \prime }}\left(z_{1}\right)/2\tilde{g}\left(z_{1}\right)\right)\left(\sigma_{z_{1}}^{2}-\sigma_{x_{1}}^{2}\right)+\left(g_{1}^{{}^{\prime \prime \prime \prime }}\left(z_{1}\right)/8\tilde{g}\left(z_{1}\right)\right){\left(\sigma_{z_{1}}^{2}-\sigma_{x_{1}}^{2}\right)}^{2}\\ {E\left[x_{1}|z_{1}\right]}_{down}=1+3T-15V+\left(g^{{}^{\prime \prime }}\left(z_{1}\right)/2\tilde{g}\left(z_{1}\right)\right)\left(\sigma_{z_{1}}^{2}-\sigma_{x_{1}}^{2}\right)+\left(g^{{}^{\prime \prime \prime \prime }}\left(z_{1}\right)/8\tilde{g}\left(z_{1}\right)\right){\left(\sigma_{z_{1}}^{2}-\sigma_{x_{1}}^{2}\right)}^{2}. \end{array}$$

Next, in order to reduce the computational complexity, we notice that the denominator of (32) (${E\left[x_{1}|z_{1}\right]}_{down}$ from (33)) can be approximated by:

(34) $${E\left[x_{1}|z_{1}\right]}_{down}≃1+\left({\tilde{g}}^{{}^{\prime \prime }}\left(z_{1}\right)/2\tilde{g}\left(z_{1}\right)\right)\left(\sigma_{z_{1}}^{2}-\sigma_{x_{1}}^{2}\right)+\left({\tilde{g}}^{{}^{\prime \prime \prime \prime }}\left(z_{1}\right)/8\tilde{g}\left(z_{1}\right)\right){\left(\sigma_{z_{1}}^{2}-\sigma_{x_{1}}^{2}\right)}^{2},$$

where ${\tilde{g}}^{{}^{\prime \prime }}\left(z_{1}\right)$ and ${\tilde{g}}^{{}^{\prime \prime \prime \prime }}\left(z_{1}\right)$ are the second and fourth derivative of $\tilde{g}\left(z_{1}\right)$ respectively. Please note that (34) is valid for the Gaussian convolutional noise pdf case. By using (32) with ${E\left[x_{1}|z_{1}\right]}_{down}$ and ${E\left[x_{1}|z_{1}\right]}_{up}$ from (34) and (33) respectively and the following derivatives:

(35) $$\begin{array}{c} k=2,4;\;K=4\\ {\tilde{g}}^{{}^{\prime }}\left(z_{1}\right)=\tilde{g}\left(z_{1}\right)\sum_{k=2}^{K}kz_{1}^{k-1}\lambda_{k}\\ {\tilde{g}}^{{}^{\prime \prime }}\left(z_{1}\right)=\tilde{g}\left(z_{1}\right){\left(\sum_{k=2}^{K}kz_{1}^{k-1}\lambda_{k}\right)}^{2}+\tilde{g}\left(z_{1}\right)\sum_{k=2}^{K}kz_{1}^{k-2}\lambda_{k}\left(k-1\right)\\ {\tilde{g}}^{{}^{\prime \prime \prime }}\left(z_{1}\right)=\tilde{g}\left(z_{1}\right){\left(\sum_{k=2}^{K}kz_{1}^{k-1}\lambda_{k}\right)}^{3}+\tilde{g}\left(z_{1}\right)3\sum_{k=2}^{K}kz_{1}^{k-2}\lambda_{k}\left(k-1\right)\sum_{k=2}^{K}kz_{1}^{k-1}\lambda_{k}\\ \tilde{g}\left(z_{1}\right)\sum_{k=2}^{K}kz_{1}^{k-3}\lambda_{k}\left(k-1\right)\left(k-2\right)\\ {\tilde{g}}^{{}^{\prime \prime \prime \prime }}\left(z_{1}\right)=3\tilde{g}\left(z_{1}\right){\left(\sum_{k=2}^{K}kz_{1}^{k-2}\lambda_{k}\left(k-1\right)\right)}^{2}+6\tilde{g}\left(z_{1}\right)\sum_{k=2}^{K}kz_{1}^{k-2}\lambda_{k}\left(k-1\right){\left(\sum_{k=2}^{K}kz_{1}^{k-1}\lambda_{k}\right)}^{2}+\\ \tilde{g}\left(z_{1}\right){\left(\sum_{k=2}^{K}kz_{1}^{k-1}\lambda_{k}\right)}^{4}+4\tilde{g}\left(z_{1}\right)\sum_{k=2}^{K}kz_{1}^{k-3}\lambda_{k}\left(k-1\right)\left(k-2\right)\sum_{k=2}^{K}kz_{1}^{k-1}\lambda_{k}+\\ \tilde{g}\left(z_{1}\right)\sum_{k=2}^{K}kz_{1}^{k-4}\lambda_{k}\left(k-1\right)\left(k-2\right)\left(k-3\right) \end{array}$$

(36) $$\begin{array}{c} {g_{1}}^{{}^{\prime \prime }}\left(z_{1}\right)=2{\tilde{g}}^{{}^{\prime }}\left(z_{1}\right)\left(3T-15V+1\right)+z_{1}{\tilde{g}}^{{}^{\prime \prime }}\left(z_{1}\right)\left(3T-15V+1\right)-z_{1}\tilde{g}\left(z_{1}\right)\left(\frac{12T}{\sigma_{p_{1}}^{2}}-\frac{90V}{\sigma_{p_{1}}^{2}}\right)\\ {g_{1}}^{{}^{\prime \prime \prime \prime }}\left(z_{1}\right)=4{\tilde{g}}^{{}^{\prime \prime \prime }}\left(z_{1}\right)\left(3T-15V+1\right)-12{\tilde{g}}^{{}^{\prime }}\left(z_{1}\right)\left(\frac{12T}{\sigma_{p_{1}}^{2}}-\frac{90V}{\sigma_{p_{1}}^{2}}\right)+\\ z_{1}\tilde{g}\left(z_{1}\right)\left(\frac{24T}{\sigma_{p_{1}}^{4}}-\frac{360V}{\sigma_{p_{1}}^{4}}\right)+z_{1}{\tilde{g}}^{{}^{\prime \prime \prime \prime }}\left(z_{1}\right)\left(3T-15V+1\right)-6z_{1}{\tilde{g}}^{{}^{\prime \prime }}\left(z_{1}\right)\left(\frac{12T}{\sigma_{p_{1}}^{2}}-\frac{90V}{\sigma_{p_{1}}^{2}}\right), \end{array}$$

the expression of $\frac{u_{1}}{f_{1}}$ from (7) is obtained. Now, by using (13), the expression from (7) is obtained. □

## 4. Simulation

In this section, we use the 16QAM input case with two different channels to show via simulation results the usefulness of our new proposed model for the convolutional noise pdf based on the GDD [17,18] and Edgeworth Expansion [15,16] compared to the Gaussian case. For equalization performance comparison, we use the MaxEnt algorithm [1], where the conditional expectation is derived by assuming the Gaussian model for the convolutional noise pdf and the source pdf is approximated with the Maximum Entropy density approximation technique [13,14] as it is done with our new proposed equalization method. Thus, the difference between the two approximated expressions for the conditional expectation ([1] and (7)) is only due to the different model used for the convolutional noise pdf. In addition, we use for the equalization performance comparison also two additional equalization methods [2,5] which we name as $MaxEnt_{BNEW}$ and $MaxEnt_{ANEW}$ respectively. These methods ([2,5]) are versions of the original MaxEnt algorithm [1] where also the convolutional noise pdf was approximated with the Gaussian model.

The equalizer’s taps for the Maximum Entropy algorithm (MaxEnt) [1] were updated according to:

(37) $$c_{l}\left[n+1\right]=c_{l}\left[n\right]-\mu_{me}Wy^{*}\left[n-l\right],$$

with:

(38) $$W=\left[\left(E\left[x_{1}|z_{1}\right]\left[\frac{\left(z_{1}\left[n\right]E\left[x_{1}|z_{1}\right]\right)}{{\langle {\left(z_{1}\right)}^{2}\rangle }_{n}}\right]+jE\left[x_{2}|z_{2}\right]\left[\frac{\left(z_{2}\left[n\right]E\left[x_{2}|z_{2}\right]\right)}{{\langle {\left(z_{2}\right)}^{2}\rangle }_{n}}\right]\right)-z\left[n\right]\right],$$

where $\mu_{me}$ is a positive step-size parameter and

(39) $$\begin{array}{c} E\left[x_{1}|z_{1}\right]=\frac{z_{1}+\frac{{\hat{g}}_{1}^{\prime \prime }\left(z_{1}\right)}{2\hat{g}\left(z_{1}\right)}\left(\sigma_{x_{1}}^{2}-\sigma_{z_{1}}^{2}\right)+\frac{{\hat{g}}_{1}^{\left(4\right)}\left(z_{1}\right)}{8\hat{g}\left(z_{1}\right)}{\left(\sigma_{x_{1}}^{2}-\sigma_{z_{1}}^{2}\right)}^{2}}{1+\frac{{\hat{g}}^{\prime \prime }\left(z_{1}\right)}{2\hat{g}\left(z_{1}\right)}\left(\sigma_{x_{1}}^{2}-\sigma_{z_{1}}^{2}\right)+\frac{{\hat{g}}^{\left(4\right)}\left(z_{1}\right)}{8\hat{g}\left(z_{1}\right)}{\left(\sigma_{x_{1}}^{2}-\sigma_{z_{1}}^{2}\right)}^{2}}\\ E\left[x_{2}|z_{2}\right]=\frac{z_{2}+\frac{{\hat{g}}_{1}^{\prime \prime }\left(z_{2}\right)}{2\hat{g}\left(z_{2}\right)}\left(\sigma_{x_{2}}^{2}-\sigma_{z_{2}}^{2}\right)+\frac{{\hat{g}}_{1}^{\left(4\right)}\left(z_{2}\right)}{8\hat{g}\left(z_{2}\right)}{\left(\sigma_{x_{2}}^{2}+\sigma_{z_{2}}^{2}\right)}^{2}}{1+\frac{{\hat{g}}^{\prime \prime }\left(z_{2}\right)}{2\hat{g}\left(z_{2}\right)}\left(\sigma_{x_{2}}^{2}-\sigma_{z_{2}}^{2}\right)+\frac{{\hat{g}}^{\left(4\right)}\left(z_{2}\right)}{8\hat{g}\left(z_{2}\right)}{\left(\sigma_{x_{2}}^{2}-\sigma_{z_{2}}^{2}\right)}^{2}}, \end{array}$$

where:

(40) $$\begin{array}{c} k=2,4;\;K=4\\ s=1,2;\;\hat{g}\left(z_{s}\right)={\left\{\exp\left(\sum_{k=2}^{k=K}\lambda_{k}x_{s}^{k}\right)\right\}}_{x_{s}=z_{s}}\\ {\hat{g}}^{\prime \prime }\left(z_{s}\right)={\left\{\frac{d^{2}}{dx_{s}^{2}}\left[\exp\left(\sum_{k=2}^{k=K}\lambda_{k}x_{s}^{k}\right)\right]\right\}}_{x_{s}=z_{s}};\;{\hat{g}}^{\left(4\right)}\left(z_{s}\right)={\left\{\frac{d^{4}}{dx_{s}^{4}}\left[\exp\left(\sum_{k=2}^{k=K}\lambda_{k}x_{s}^{k}\right)\right]\right\}}_{x_{s}=z_{s}}\\ {\hat{g}}_{1}^{\prime \prime }\left(z_{s}\right)={\left\{\frac{d^{2}}{dx_{s}^{2}}\left[x_{s}\exp\left(\sum_{k=2}^{k=K}\lambda_{k}x_{s}^{k}\right)\right]\right\}}_{x_{s}=z_{s}};\;{\hat{g}}_{1}^{\left(4\right)}\left(z_{s}\right)={\left\{\frac{d^{4}}{dx_{s}^{4}}\left[x_{s}\exp\left(\sum_{k=2}^{k=K}\lambda_{k}x_{s}^{k}\right)\right]\right\}}_{x_{s}=z_{s}} \end{array}$$

and $\sigma_{x_{1}}^{2}$,$\sigma_{x_{2}}^{2}$ are the variances of the real and imaginary parts of the source signal respectively. The variances of the real and imaginary parts of the equalized output are defined as $\sigma_{z_{1}}^{2}$ and $\sigma_{z_{2}}^{2}$ respectively and estimated by [1]:

(41) $$\langle z_{s}^{2}\rangle =\left(1-\beta_{me}\right){\langle z_{s}^{2}\rangle }_{n-1}+\beta_{me}{\left(z_{s}\right)}_{n}^{2},$$

where   stands for the estimated expectation, ${\langle z_{s}^{2}\rangle }_{0}>0$, *l* stands for the l-th tap of the equalizer and $\beta_{me}$ is a positive step size parameter. The Lagrange multipliers $\lambda_{k}$ from (40) are given in (11). According to [1] the equalizer’s taps are updated only if ${\hat{N}}_{s}>\varepsilon$, where ε is a small positive parameter and ${\hat{N}}_{s}=1+\frac{{\hat{g}}^{\prime \prime }\left(z_{1}\right)}{2\hat{g}\left(z_{1}\right)}\left(\sigma_{x_{s}}^{2}-\sigma_{z_{s}}^{2}\right)+\frac{{\hat{g}}^{\left(4\right)}\left(z_{1}\right)}{8\hat{g}\left(z_{1}\right)}{\left(\sigma_{x_{s}}^{2}-\sigma_{z_{s}}^{2}\right)}^{2}$. In the following we denote our new proposed equalization method based on the GDD [17,18] as GDD were the equalizer’s taps are updated according to:

(42) $$c_{l}\left[n+1\right]=c_{l}\left[n\right]-\mu Wy^{*}\left[n-l\right],$$

where μ is a positive step size parameter and *W* is given in (38) with:

(43) $$E\left[x_{1}|z_{1}\right]=\frac{u_{1}}{f_{1}};\;\;E\left[x_{2}|z_{2}\right]=\frac{u_{2}}{f_{2}},$$

where $\frac{u_{1}}{f_{1}}$ and $\frac{u_{2}}{f_{2}}$ are given in (7). The variances of the real and imaginary parts of the convolutional noise ($\sigma_{p_{1}}^{2}$ and $\sigma_{p_{2}}^{2}$) are given by:

(44) $$\begin{array}{c} s=1,2\\ \sigma_{p_{s}}^{2}=\sigma_{z_{s}}^{2}-\sigma_{x_{s}}^{2}\\ \langle z_{s}^{2}\rangle =\left(1-\beta \right){\langle z_{s}^{2}\rangle }_{n-1}+\beta {\left(z_{s}\right)}_{n}^{2}, \end{array}$$

where β is a positive step size parameter. It should be pointed out that the equalizer’s taps related to the GGD algorithm are updated only when $f_{1}>\varepsilon$ and $f_{2}>\varepsilon$ similar to the MaxEnt algorithm. The equalizer’s taps related to the $MaxEnt_{ANEW}$ algorithm are updated according to [5]:

(45) $${\tilde{c}}_{l}\left[n+1\right]=c_{l}\left[n\right]-\mu_{ANEW}Wy^{*}\left[n-l\right],$$

where $\mu_{ANEW}$ is a positive step size parameter and *W* is given in (38) with:

(46) $$\begin{array}{c} E\left[x_{1}|z_{1}\right]≃\left(1+\left(\varepsilon_{0}^{1}+\varepsilon_{2}^{1}z_{1}^{2}+\varepsilon_{4}^{1}z_{1}^{4}\right)+\frac{1}{2}{\left(\varepsilon_{0}^{1}+\varepsilon_{2}^{1}z_{1}^{2}+\varepsilon_{4}^{1}z_{1}^{4}\right)}^{2}\right)\left(z_{1}+\frac{\sigma_{p_{1}}^{2}}{2}\frac{g_{1}^{{}^{\prime \prime }}\left(z_{1}\right)}{g(z_{1})}+\frac{{\left(\sigma_{p_{1}}^{2}\right)}^{2}}{8}\frac{g_{1}^{{}^{{}^{\prime \prime \prime \prime }}}\left(z_{1}\right)}{g(z_{1})}\right)\\ E\left[x_{2}|z_{2}\right]≃\left(1+\left(\varepsilon_{0}^{2}+\varepsilon_{2}^{2}z_{2}^{2}+\varepsilon_{4}^{2}z_{2}^{4}\right)+\frac{1}{2}{\left(\varepsilon_{0}^{2}+\varepsilon_{2}^{2}z_{2}^{2}+\varepsilon_{4}^{2}z_{2}^{4}\right)}^{2}\right)\left(z_{2}+\frac{\sigma_{p_{2}}^{2}}{2}\frac{g_{1}^{{}^{\prime \prime }}\left(z_{2}\right)}{g(z_{2})}+\frac{{\left(\sigma_{p_{2}}^{2}\right)}^{2}}{8}\frac{g_{1}^{{}^{{}^{\prime \prime \prime \prime }}}\left(z_{2}\right)}{g(z_{2})}\right),\\ where:\\ s=1,2\\ \frac{g_{1}^{{}^{\prime \prime }}\left(z_{s}\right)}{g(z_{s})}=2z_{s}\left(8z_{s}^{6}\lambda_{4}^{2}+8z_{s}^{4}\lambda_{2}\lambda_{4}+2z_{s}^{2}\lambda_{2}^{2}+10z_{s}^{2}\lambda_{4}+3\lambda_{2}\right)\\ \frac{g_{1}^{{}^{{}^{\prime \prime \prime \prime }}}\left(z_{s}\right)}{g(z_{s})}=4z_{s}\left(64z_{s}^{12}\lambda_{4}^{4}+128z_{s}^{10}\lambda_{2}\lambda_{4}^{3}+96z_{s}^{8}\lambda_{2}^{2}\lambda_{4}^{2}+352z_{s}^{8}\lambda_{4}^{3}+32z_{s}^{6}\lambda_{2}^{3}\lambda_{4}+432z_{s}^{6}\lambda_{2}\lambda_{4}^{2}+\right.\\ \left.4z_{s}^{4}\lambda_{2}^{4}+168z_{s}^{4}\lambda_{2}^{2}\lambda_{4}+348z_{s}^{4}\lambda_{4}^{2}+20z_{s}^{2}\lambda_{2}^{3}+180z_{s}^{2}\lambda_{2}\lambda_{4}+15\lambda_{2}^{2}+30\lambda_{4}\right)\\ \sigma_{p_{s}}^{2}=\sigma_{z_{s}}^{2}-\sigma_{x_{s}}^{2} \end{array}$$

and

(47) $$\sigma_{x_{s}}^{2}=E\left[x_{s}^{2}\right].$$

According to [5]:

(48) $$\sigma_{z_{s}}^{2}=E\left[z_{s}^{2}\right]$$

and given by:

(49) $$\langle z_{s}^{2}\rangle =\left(1-\beta_{ANEW}\right){\langle z_{s}^{2}\rangle }_{n-1}+\beta_{ANEW}{\left(z_{s}\right)}_{n}^{2},$$

where ${\langle z_{s}^{2}\rangle }_{0}>0$, $\beta_{ANEW}$ and $\mu_{ANEW}$ are positive step size parameters. $\varepsilon_{0}^{s}$, $\varepsilon_{2}^{s}$, $\varepsilon_{4}^{s}$, $\lambda_{2}$ and $\lambda_{4}$ were set according to [5] as

(50) $$\begin{array}{c} \varepsilon_{0}^{s}=-2\lambda_{2}\sigma_{p_{s}}^{2};\;\;\varepsilon_{2}^{s}=-\sigma_{p_{s}}^{2}\left(4\lambda_{2}^{2}+12\lambda_{4}\right);\;\;\varepsilon_{4}^{s}=-16\lambda_{2}\lambda_{4}\sigma_{p_{s}}^{2} \end{array}$$

(51) $$\begin{array}{c} \lambda_{2}≃\frac{1}{40{\bar{m}}_{2}\left(20736{\bar{m}}_{4}^{2}+1280{\bar{m}}_{2}{\bar{m}}_{6}\right)}\left(41472{\bar{m}}_{4}^{2}+2560{\bar{m}}_{2}{\bar{m}}_{6}-144{\bar{m}}_{4}\left(480{\bar{m}}_{2}^{2}+288{\bar{m}}_{4}\right)\right)\\ \lambda_{4}≃\frac{1}{20736{\bar{m}}_{4}^{2}+1280{\bar{m}}_{2}{\bar{m}}_{6}}\left(480{\bar{m}}_{2}^{2}+288{\bar{m}}_{4}\right), \end{array}$$

where

(52) $$E\left[x_{1}^{G}\right]={\bar{m}}_{G}.$$

In order to get equalization gain of one, the following gain control was used according to [5]:

(53) $$c_{l}\left[n\right]=\frac{{\tilde{c}}_{l}}{\sqrt{\sum_{l}{\left|{\tilde{c}}_{l}\right|}^{2}}},$$

where $c_{l}\left[n\right]$ is the vector of taps after iteration and $c_{l}\left[0\right]$ is some reasonable initial guess. The equalizer’s taps related to the $MaxEnt_{BNEW}$ algorithm are updated according to [2]:

(54) $${\tilde{c}}_{l}\left[n+1\right]=c_{l}\left[n\right]-\mu_{BNEW}Wy^{*}\left[n-l\right],$$

where $\mu_{BNEW}$ is a positive step size parameter and *W* is given in (38) with:

(55) $$\begin{array}{c} E\left[x_{1}|z_{1}\right]=\frac{z_{1}+\frac{{\hat{g}}_{1}^{\prime \prime }\left(z_{1}\right)}{2\hat{g}\left(z_{1}\right)}\sigma_{p_{1}}^{2}}{1+\frac{{\hat{g}}^{\prime \prime }\left(z_{1}\right)}{2\hat{g}\left(z_{1}\right)}\sigma_{p_{1}}^{2}}\\ E\left[x_{2}|z_{2}\right]=\frac{z_{2}+\frac{{\hat{g}}_{1}^{\prime \prime }\left(z_{2}\right)}{2\hat{g}\left(z_{2}\right)}\sigma_{p_{2}}^{2}}{1+\frac{{\hat{g}}^{\prime \prime }\left(z_{2}\right)}{2\hat{g}\left(z_{2}\right)}\sigma_{p_{2}}^{2}},\\ where:\\ s=1,2\\ \frac{{\hat{g}}_{1}^{{}^{\prime \prime }}\left(z_{s}\right)}{2\hat{g}\left(z_{s}\right)}=z_{s}\left(8z_{s}^{6}\lambda_{4}^{2}+8z_{s}^{4}\lambda_{2}\lambda_{4}+2z_{s}^{2}\lambda_{2}^{2}+10z_{s}^{2}\lambda_{4}+3\lambda_{2}\right)\\ \frac{{\hat{g}}^{{}^{\prime \prime }}\left(z_{s}\right)}{2\hat{g}\left(z_{s}\right)}=8z_{s}^{6}\lambda_{4}^{2}+8z_{s}^{4}\lambda_{2}\lambda_{4}+2z_{s}^{2}\lambda_{2}^{2}+6z_{s}^{2}\lambda_{4}+\lambda_{2}\\ \sigma_{p_{s}}^{2}=\sigma_{z_{s}}^{2}-\sigma_{x_{s}}^{2} \end{array}$$

(56) $$\begin{array}{c} \lambda_{2}=\frac{1}{4{\hat{m}}_{2}\left(64{\hat{m}}_{4}^{2}-64{\hat{m}}_{2}{\hat{m}}_{6}\right)}\left(64{\hat{m}}_{2}{\hat{m}}_{6}-64{\hat{m}}_{4}^{2}+8{\hat{m}}_{4}\left(8{\hat{m}}_{4}-24{\hat{m}}_{2}^{2}\right)\right)\\ \lambda_{4}=-\frac{1}{64{\hat{m}}_{4}^{2}-64{\hat{m}}_{2}{\hat{m}}_{6}}\left(8{\hat{m}}_{4}-24{\hat{m}}_{2}^{2}\right)\\ \;\;\mathrm{with}\\ {\hat{m}}_{2}={\bar{m}}_{2}\left(1+\frac{1}{SNR\sum_{k=0}^{R-1}{\left|h_{k}\right|}^{2}}\right)\\ {\hat{m}}_{4}={\bar{m}}_{2}^{2}\left(\frac{3}{{\left(SNR\sum_{k=0}^{R-1}{\left|h_{k}\right|}^{2}\right)}^{2}}+\frac{6}{SNR\sum_{k=0}^{R-1}{\left|h_{k}\right|}^{2}}+\frac{{\bar{m}}_{4}}{{\bar{m}}_{2}^{2}}\right)\\ {\hat{m}}_{6}={\bar{m}}_{2}^{3}\left(\frac{15}{{\left(SNR\sum_{k=0}^{R-1}{\left|h_{k}\right|}^{2}\right)}^{3}}+\frac{45}{{\left(SNR\sum_{k=0}^{R-1}{\left|h_{k}\right|}^{2}\right)}^{2}}+\frac{15}{SNR\sum_{k=0}^{R-1}{\left|h_{k}\right|}^{2}}\frac{{\bar{m}}_{4}}{{\bar{m}}_{2}^{2}}+\frac{{\bar{m}}_{6}}{{\bar{m}}_{2}^{3}}\right), \end{array}$$

where

(57) $$SNR=\frac{{\bar{m}}_{2}}{\sigma_{w_{r}}^{2}}.$$

$\sigma_{z_{s}}^{2}$ was estimated by

(58) $$\langle z_{s}^{2}\rangle =\left(1-\beta_{BNEW}\right){\langle z_{s}^{2}\rangle }_{n-1}+\beta_{BNEW}{\left(z_{s}\right)}_{n}^{2},$$

where ${\langle z_{s}^{2}\rangle }_{0}>0$, $\beta_{BNEW}$ and $\mu_{BNEW}$ are positive step size parameters. The equalizer’s taps in (54) were updated only if ${\hat{N}}_{s}>\varepsilon_{1}$, where $\varepsilon_{1}$ is a small positive parameter and

(59) $${\hat{N}}_{s}=1+\frac{{\hat{g}}^{\prime \prime }\left(z_{s}\right)}{2\hat{g}\left(z_{s}\right)}\sigma_{p_{s}}^{2}.$$

In addition, the gain control was applied according to (53).

Two different channels were considered:

- Easy channel case, **Channel1** (initial ISI = 0.44): The channel parameters were determined according to [6]: $h_{n}=\{0\;$ for n<0;−0.4 for $\;n=0;\;0.84\times 0.4^{n-1}\;$ for n>0}
- Hard channel case, **Channel2** (initial ISI = 1.402): The channel parameters were taken according to [22]: $h_{n}=\left(0.2258,0.5161,0.6452,0.5161\right)$

**Figure 2:**
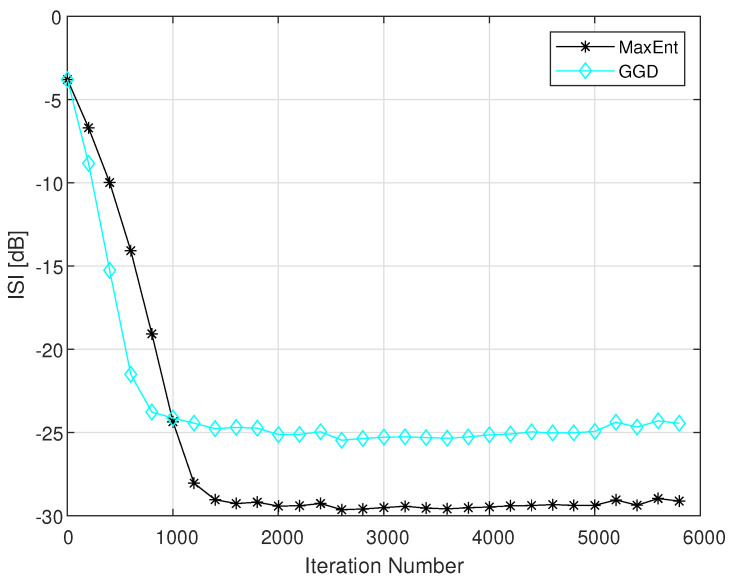
Equalization performance comparison between the GGD and MaxEnt methods for a 16QAM input going through channel1. The averaged result were obtained in 100 Monte Carlo trials for a SNR of 26 dB. The step size parameters were set to: $\mu =6\times 10^{-4},\beta =1\times 10^{-4},\mu_{me}=3\times 10^{-4},\beta_{me}=2\times 10^{-4}$. In addition we set: ε=0.

For Channel1 and Channel4, we used an equalizer with 13 and 21 taps respectively. In the simulation, the equalizers were initialized by setting the center tap equal to one and all others to zero [1]. The step size parameters μ, β, $\mu_{me}$ and $\beta_{me}$, were chosen for fast convergence with low steady state ISI, where the values for $\mu_{me}$ and $\beta_{me}$ were taken from [1]. Figure 2 shows the simulated ISI as a function of the iteration number of our new proposed algorithm (GGD), compared to the MaxEnt method [1], for the 16QAM input and Channel1 case for signal-to noise-ratio (SNR) of 26 dB according to [1].

**Figure 3:**
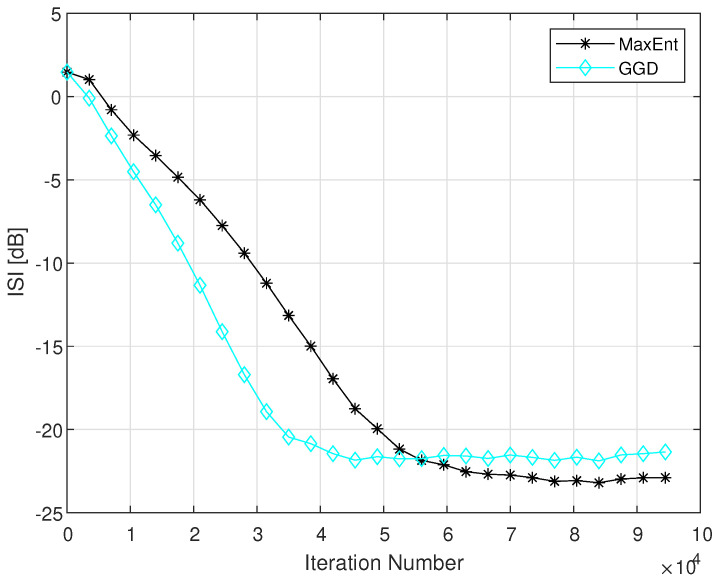
Equalization performance comparison between the GGD and MaxEnt methods for a 16QAM input going through channel4. The averaged result were obtained in 50 Monte Carlo trials for a SNR of 30 dB. The step size parameters were set to: $\mu =3\times 10^{-4},\beta =2\times 10^{-6},\mu_{me}=2\times 10^{-4},\beta_{me}=2\times 10^{-6}$. In addition we set: ε=0.5.

Please note that the main purpose of a blind adaptive equalizer is to be as fast as possible, a residual ISI that is low enough for sending the equalized output sequence to the decision device to get reliable decisions on that input data. Reliable decisions can be done on the equalized output sequence when the equalizer leaves the system with a residual ISI that is lower than −16 dB. According to Figure 2, the new algorithm (GGD) achieves the residual ISI of −16 dB faster than the MaxEnt algorithm [1]. Thus, the GGD has a faster convergence rate compared to the MaxEnt [1] method, which means that the equalized output sequence can be send earlier to the decision device with the GGD algorithm compared with the MaxEnt method [1]. Figure 3 shows the simulated ISI as a function of the iteration number of our new proposed algorithm (GGD), compared to the MaxEnt method [1], for the 16QAM input and Channel4 case for SNR of 30 dB according to [1].

According to Figure 3, the GGD algorithm reaches the residual ISI of −16 dB faster by approximately of 15,000 symbols than the MaxEnt [1] algorithm does while leaving the system with approximately the same residual ISI at the convergence state compared with the MaxEnt [1] method.

**Figure 4:**
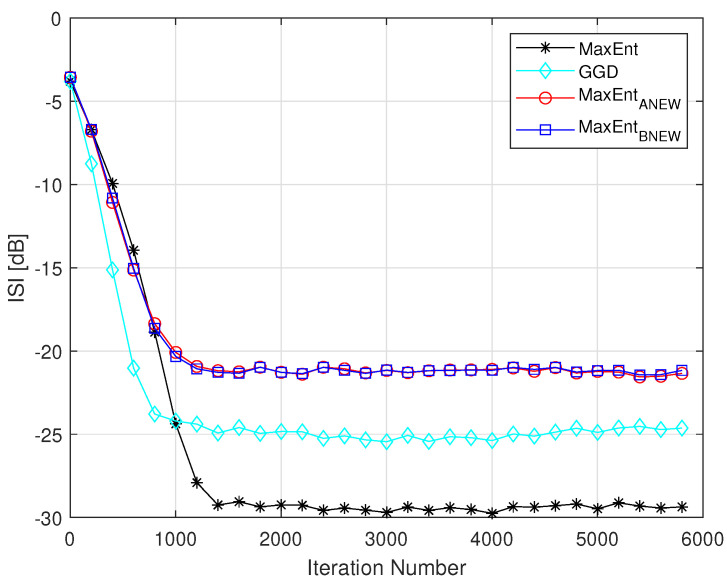
Equalization performance comparison between the GGD, MaxEnt, $MaxEnt_{ANEW}$ and $MaxEnt_{BNEW}$ methods for a 16QAM input going through channel1. The averaged result were obtained in 100 Monte Carlo trials for a SNR of 26 dB. The step size parameters were set to: $\mu =6\times 10^{-4},\beta =1\times 10^{-4},\mu_{me}=3\times 10^{-4},\beta_{me}=2\times 10^{-4},\mu_{ANEW}=3\times 10^{-4},\beta_{ANEW}=2\times 10^{-5},\mu_{BNEW}=3\times 10^{-4},\beta_{BNEW}=2\times 10^{-4}$. In addition we set: $\varepsilon =0,\varepsilon_{1}=0.5$.

It should be pointed out that the equalization performance obtained with the GDD algorithm are very similar to those obtained in [4] where the Edgeworth Expansion up to order six was used for approximating the convolutional noise pdf. However, in [4], two additional step parameters were needed in the deconvolution process. Those step size parameters are channel dependent which are not needed in the GDD algorithm. Thus, the GDD algorithm is preferable over the algorithm proposed in [4]. The GDD algorithm has also improved equalization performance for the hard channel case (Channel2) compared to the equalization method proposed in [3] where the Maximum Entropy density approximation technique [13,14] was used for approximating the convolutional noise pdf with Lagrange multipliers up to order four. Please note that according to [3], the MaxEnt method [1] and the equalization algorithm proposed in [3] have the same equalization performance for the hard channel case (Channel2). Figure 4 shows the simulated ISI as a function of the iteration number of our new proposed algorithm (GGD), compared to the MaxEnt method [1], to the $MaxEnt_{ANEW}$ method [5] and to the $MaxEnt_{BNEW}$ method [2] for the 16QAM input and Channel1 case for SNR of 26 dB. According to Figure 4, the GGD algorithm has improved equalization performance from the residual ISI and convergence time point of view compared to the $MaxEnt_{ANEW}$ [5] and $MaxEnt_{BNEW}$ [2] methods. From the residual ISI point of view, the improvement is approximately 4 dB while the improvement in the convergence time is approximately third of the convergence time achieved by the equalization methods presented in [2,5].

Although the GGD algorithm was obtained for the 16QAM constellation input, it can be extended to other two independent quadrature carrier inputs with Lagrange multiplier up to order four, by having just another function for β (9) that fits the new input constellation case. In addition, if more Lagrange multipliers are needed than only four for approximating properly the input sequence pdf, (7) should be used with k=2,4,6,...K and the Lagrange multipliers should be calculated as given in [1] for the general order case.

## 5. Conclusions

In this paper, the blind adaptive deconvolution problem was considered, where the GGD function and the Edgeworth Expansion up to order six were applied for approximating the convolutional noise pdf for the 16 QAM input case. A new closed-form approximated expression was derived for the conditional expectation that led to a new blind adaptive equalization method. This new proposed algorithm does not need additional predefined parameters that are channel dependent like the literature known blind adaptive equalization method based on the conditional expectation expression where the convolutional noise pdf was approximated with the Edgeworth Expansion up to order six. Simulation results demonstrated that improved equalization performance is obtained with our new proposed equalization method based on the new model for the convolutional noise pdf compared to the original Maximum Entropy algorithm and to the two recently obtained versions of the original Maximum Entropy algorithm for the easy channel and high SNR case. Since the original Maximum Entropy algorithm has the same equalization performance for the hard channel case as the equalization method based on the conditional expectation expression where the convolutional noise pdf was approximated with the Maximum Entropy density technique, the new proposed method has also improved equalization performance for the hard channel case compared with this equalization method. This paper demonstrated that improved equalization performance can be obtained if a proper approximation is applied for the convolutional noise pdf in the calculation for the expression of the conditional expectation via Bayes rules. The new proposed algorithm is valid only for the high SNR case due to the fact that the noise was not taken into account in our derivations. Please note that the original Maximum Entropy algorithm and the two equalization methods based on the conditional expectation via Bayes rules, where the convolutional noise pdf was approximated with the Maximum Entropy density technique and with the Edgeworth Expansion approach, are valid also only for the high SNR case.

## Data Availability

All the required data is given in the article.
